# Biologics in Asthma: A Molecular Perspective to Precision Medicine

**DOI:** 10.3389/fphar.2021.793409

**Published:** 2022-01-19

**Authors:** Brittany Salter, Paige Lacy, Manali Mukherjee

**Affiliations:** ^1^ Division of Respirology, Department of Medicine, McMaster University, Hamilton, ON, Canada; ^2^ Firestone Institute for Respiratory Health, St. Joseph’s Healthcare, Hamilton, ON, Canada; ^3^ Department of Medicine, University of Alberta, Edmonton, AB, Canada

**Keywords:** severe asthma, monoclonal antibodies, T2 inflammation, eosinophil, treatment response

## Abstract

Recent developments in therapeutic strategies have provided alternatives to corticosteroids as the cornerstone treatment for managing airway inflammation in asthma. The past two decades have witnessed a tremendous boost in the development of anti-cytokine monoclonal antibody (mAb) therapies for the management of severe asthma. Novel biologics that target eosinophilic inflammation (or type 2, T2 inflammation) have been the most successful at treating asthma symptoms, though there are a few in the drug development pipeline for treating non-eosinophilic or T2-low asthma. There has been significant improvement in clinical outcomes for asthmatics treated with currently available monoclonal antibodies (mAbs), including anti-immunoglobulin (Ig) E, anti-interleukin (IL)-4 receptor α subunit, anti-IL-5, anti-IL-5Rα, anti-IL-6, anti-IL-33, and anti-thymic stromal lymphopoietin (TSLP). Despite these initiatives in precision medicine for asthma therapy, a significant disease burden remains, as evident from modest reduction of exacerbation rates, i.e., approximately 40–60%. There are numerous studies that highlight predictors of good responses to these biologics, but few have focused on those who fail to respond adequately despite targeted treatment. Phenotyping asthmatics based on blood eosinophils is proving to be inadequate for choosing the right drug for the right patient. It is therefore pertinent to understand the underlying immunology, and perhaps, carry out immune endotyping of patients before prescribing appropriate drugs. This review summarizes the immunology of asthma, the cytokines or receptors currently targeted, the possible mechanisms of sub-optimal responses, and the importance of determining the immune make-up of individual patients prior to prescribing mAb therapy, in the age of precision medicine for asthma.

## 1 Introduction

Asthma is defined by reversible airflow obstruction, hyperresponsiveness, and inflammation, that manifests as wheeze, dyspnea, and cough. Despite a wide array of treatments available for asthma, 5–10% of patients have poor response to inhaled corticosteroids, and remain on high doses of systemic corticosteroids (Heffler et al., 2019). At first glance, this may seem like a non-significant percentile; however this subgroup contributes substantially to the economical disease burden, accounting for 56 billion US dollars annually, due to frequent exacerbations with need for acute care (Barnett and Nurmagambetov, 2011). Current clinical guidelines for asthma diagnosis include assessment of lung function through spirometry with or without a bronchoprovocation challenge to quantify hyperresponsiveness. Interestingly, despite airway inflammation being a hallmark feature of asthma, it is not a requirement for asthma diagnosis, but instead helps to stratify disease severity. As a whole, the current tests we have do not account for the vast immunological heterogeneity of asthma.

We have seen great strides over the past 2 decades with respect to the development of alternative therapies to corticosteroids. The era of monoclonal antibodies (mAbs) targeting receptors and cytokines involved in the pathogenesis of asthma has emerged in severe asthma management. Although we have witnessed significant improvement in clinical outcomes for severe asthmatics treated with currently available mAbs, there still remains a proportion of patients with refractory disease. Biologic therapy has used biomarkers to phenotype patients and identify those who would benefit most from therapy, using blood eosinophils, serum total IgE and periostin, and fraction of excreted nitric oxide (FeNO). Unfortunately, these biomarkers fail to reflect the complexity of underlying inflammatory endotypes, and are proving to be inadequate for not only choosing the right drug for the right patient, but also monitoring response to treatment. It has been clear that inflammation in severe asthma is not always characterized by the presence of eosinophilia. We need to pay closer attention to the patients who fail to respond to mAbs to learn lessons on how to better individualize treatment. Immunological endotyping has been proposed as a potential tool to curtail treatment for individual patient’s and needs to be further studied. This review will summarize the immunology of asthma, the cytokines or receptors currently targeted, and potential mechanisms of sub-optimal responses.

## 2 Airway Inflammation in Asthma

Asthma was initially categorized into two simple phenotypes of allergic and non-allergic disease, however over time our understanding of disease pathogenesis has expanded, and we now categorize phenotypes based on underlying inflammatory-based mechanisms (neutrophilic, eosinophilic, mixed, and pauci-granulocytic). There is evidence to suggest that even with each inflammatory phenotype there is a great deal of heterogeneity, with several different immune endotypes contributing to the overlying inflammation. Broadly, there are two asthma endotypes characterized as type 2 (T2) high and T2 low inflammation. The T2-high endotype, defined by a T2 cytokine response (IL-4, IL-5, and IL-13), is the most common endotype and the most well understood.

In order to individualize treatment, a patient’s asthma endotype must be identified and fortunately, genomics has emerged as a powerful tool for diagnosis. In severe asthma, three transcriptome-associated clusters (TACs) have been identified, including TAC 1 (*IL-33R*, *CCR3, TSLPR*), TAC2 (*interferon-, tumour necrosis factor alpha-*, and *inflammasome-associated genes*), and TAC3 (*genes of metabolic pathways, ubiquitination and mitochondrial function*). TAC1 has the highest enrichment of gene signatures for IL-13/Th2-high and innate lymphoid cell type 2 (ILC2) inflammation, along with the highest sputum and blood eosinophils and serum periostin. Furthermore, this cluster has oral corticosteroid (OCS)-dependency, frequent exacerbations, and severe airflow obstruction. Conversely, TAC2 has high sputum neutrophils and TAC3 has normal to high sputum eosinophilia and better preserved FEV_1_, with the least OCS-dependency. As such, in the setting of severe asthma, three unique clusters of gene expression have been identified, further demonstrating the heterogeneity of endotypes within each inflammatory phenotype.

### 2.1 Type 2 Inflammation

T2-high inflammation develops in response to cross-talk between innate and adaptive immune responses. Allergic asthma is triggered by inhaled allergens that are taken up within the airways by antigen presenting cells, including dendritic cells. These cells go on to process aeroallergens and present antigen peptides on their cell surface via the HLA class II molecule of the major histocompatibility complex (MHC Class II) within lymph nodes. MHC class II interacts with the T cell receptor (TCR) of naive CD4^+^ T cells, resulting in polarization towards the T helper 2 (Th2) lineage. Polarization is, in part, driven by IL-4, produced by neighbouring mast cells and basophils.

Once Th2 cells have matured, they migrate to the airways where further antigen exposure results in TCR-antigen binding and prompts Th2 cells to release T2 cytokines including IL-4, IL-5, and IL-13, leading to downstream airway inflammation (Hammad and Lambrecht, 2021). IL-4 and IL-13 induce Ig class switching of B cells to produce IgE, which has the capacity to bind to and activate high-affinity Fc**ε**R1 receptors on mast cells and basophils. After initial sensitization, re-exposure to allergen results in IgE crosslinking with Fc**ε**R1 receptors, leading to mast cell and basophil degranulation of histamine, leukotrienes, and prostaglandins, which go on to promote bronchoconstriction.

This aforementioned adaptive immune system, is also triggered by upstream innate processes. Inhaled antigens interact with airway epithelium, resulting in production of alarmins including, thymic stromal lymphopoietin (TSLP), IL-25, and IL-33 (Hammad and Lambrecht, 2021). Collectively, these alarmins promote the release of cytokines from Th2 cells, basophils, mast cells, and ILC2s (Salter et al., 2019). Similar to Th2 cells, ILC2s are potent promoters of T2-high inflammation, through production of IL-5 and IL-13 (Salter et al., 2019). In addition, basophils and mast cells, have been identified as potent sources of IL-4 and IL-13 (Bao and Reinhardt, 2015). With respect to IL-13, this cytokine also plays a role in inducing mucus production, airway remodeling, and hyperresponsiveness. In particular, for quite some time the spotlight has been on IL-5, for its an important role in asthma. This eosinophil-maturing cytokine is produced not only by Th2 cells, but also granulocytes and ILC2s. The biologic effects of IL-5 are mediated through interaction with IL-5Rα and a non-specific beta chain heterodimer, recognized by IL-3 and GM-CSF (Rossjohn et al., 2000). When IL-5 is present it binds to IL-5Rα and drives formation of a functional IL-5Rα/β chain receptor complex, that promotes activation of an intricate network of signaling pathways (Johanson et al., 1995).

IL-5Rα is highly expressed on eosinophils (Varricchi et al., 2016) and the interaction between IL-5 and IL-5Rα results in downstream activation of intracellular signaling proteins JAK2 and STAT 1, 3, and 5, which in turn stimulate transcriptional factors involved in eosinophil proliferation (Pazdrak et al., 1995). JAK2 is also involved in the inhibition of eosinophil apoptosis through the active cooperation with Lyn and Raf-1 kinases (Pazdrak et al., 1995; Schwartz et al., 2015). Other signal transduction molecules that are activated by IL-5 include phosphoinositide 3-kinase (PI3K) and mitogen-activated protein kinases (MAPK). Through activation of extracellular signal-regulated kinases (ERK)1/2 and protein kinase C (PKC), PI3K mediates IL-5-induced interaction of eosinophils with intracellular adhesion molecule-1 (ICAM-1) (Sano et al., 2005). The Ras-Raf-1-mediated activation of the ERK subfamily of MAPK drives c-fos gene transcription, which is involved in promoting cell maturation, survival, and proliferation (Adachi et al., 2000). Lastly, through a NF-kB-dependent mechanism, p38 MAPK up-regulates eosinophil pro-inflammatory cytokine production (Adachi et al., 2000). IL-5 is responsible for the activation of many integral functions of eosinophils, including maturation, accumulation and activation, and action depends on the interaction with IL-5Rα, mAbs have been developed against IL-5 and IL-5Rα.

Eosinophils exert their effects on the airways through degranulation (principally piecemeal degranulation) including the release of free intact eosinophilic granules (FEGs), which produce tissue-damaging eosinophil granule proteins, including major basic protein (MBP), eosinophilia cationic protein (ECP), eosinophilic-derived neurotoxin (EDN), and eosinophil peroxidase (EPX) (Hogan et al., 2008). Eosinophils also release extracellular DNA deposits that form a web-like structure called eosinophilic extracellular traps (EETs) through a process called ETosis (Mukherjee et al., 2018a). EETs have an autocrine effect on promoting eosinophil degranulation and inducing epithelial cells to produce IL-6 and IL-8 (Mukherjee et al., 2018a). Collectively, the aforementioned mediators contribute to airway remodeling, airway hyperreactivity, and increased mucus production.

### 2.2 Type 1 Inflammation

T2-low inflammation has emerged as another pathway that results in asthma pathogenesis. Pattern recognition receptors (PRRs) on surface of airway epithelial cells, granulocytes, dendritic cells, and T cells, act to recognize danger-associated molecular patterns (DAMPs) and pathogen-associated molecular patterns (PAMPs) and induce downstream mediator release. Activation of the PRR called nucleotide-binding oligomerization domain-like receptor (NLR) results in stimulation of inflammasomes, which are multimolecular signaling platforms that act as critical inducers of host defense (Elliott and Sutterwala, 2015). In particular, the NLRP3 inflammasome is one of the five major inflammosomes that induces secretion of IL-1β (Elliott and Sutterwala, 2015). This secretion is mediated through caspase-1, which cleaves IL-1β into its secretory isoform. IL-1β plays a role in Th17 differentiation and IL-17 production. IL-17 acts as an important mediator of neutrophilic inflammation and is elevated in severe asthmatics with frequent exacerbations (Ricciardolo et al., 2017). The inflammasome promotes pyroptosis, a form of lytic cell death. NLRP3, caspase-1, and IL-1β are increased in sputa of severe asthmatics and correlate with disease severity (Simpson et al., 2014; Kim et al., 2017). Neutrophils have been proposed to play a role in activation of the inflammasome (Wright et al., 2016). Neutrophil-derived extracellular DNA (eDNA) is released in a web-like structure to form neutrophil extracellular traps (NETs), in a process known as NETosis, which can be induced by infectious and non-infectious conditions. The presence of airway neutrophilia and NETosis results in inflammasome activation, leading to promotion of Th17-mediated inflammation. Although there are emerging biologic therapies that target T2-low inflammation, the overall identification of this endotype and use of biomarkers to monitor treatment response remains largely unknown.

## 3 Currently Available Biological Therapy and Suboptimal Responses in Severe Asthma

**FIGURE 1:**
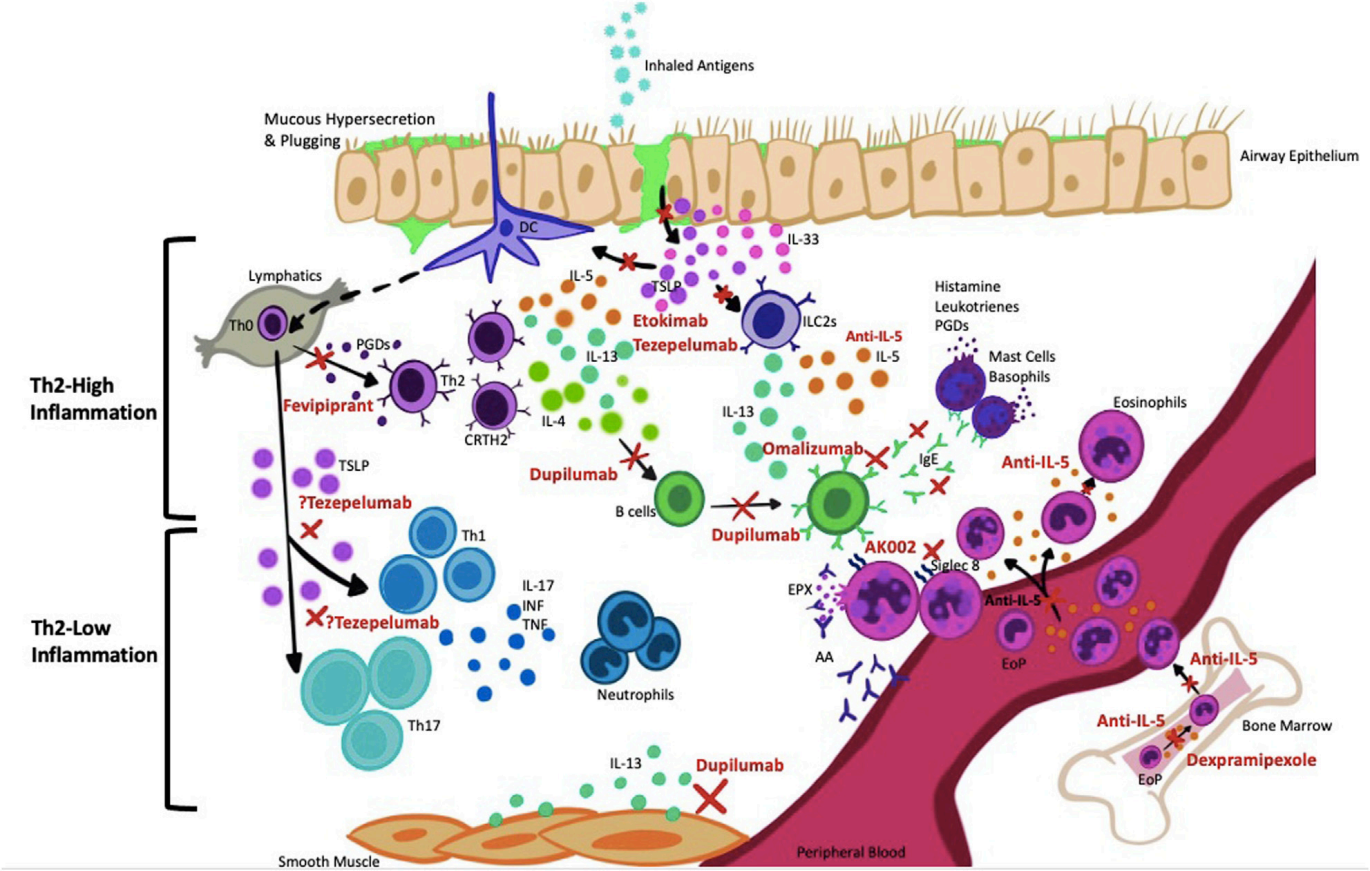
Targets of current therapy in severe asthma. There are numerous targets that have been identified for the management of severe asthma. In particular, the differentiation of eosinophil progenitors (EoP) into eosinophils, and subsequent activation of eosinophils can be targeted by anti-Siglec-8 (AK002), dexpramipexole, as well as anti-IL-5 therapies. The secretion of alarmin cytokines (TSLP, IL-33) from epithelial cells and activation of downstream ILC2s and Th2 cells can be inhibited by anti-TSLP and anti-IL-33 agents, such as tezepelumab and etokimab, respectively. The downstream action of Th2 cytokines, such as IL-4 and IL-13, produced primarily from Th2 cells, ILC2s and basophils can be inhibited by dupilumab. The cross-linking of IgE on FcεR1 receptors on mast cells and basophils can be inhibited by omalizumab, and thus prevent degranulation of leukotrienes, histamine, and prostaglandins (PGDs). PGDs play an important role in binding to CRTH2 on ILC2s and Th2 cells, promoting their migration and activation within the airways. This can be targeted by anti-CRTH2 agents such as fevipiprant. There are few identified targets for Th2-low inflammation but anti-TSLP is a potential biologic acts on this pathway. Abbreviations: AA: Autoantibodies; EoP: Eosinophil Progenitors; ILC2s: Innate Lymphoid Type 2 Cells; INF: Interferon; PGDs: Prostaglandins; TNF: Tumor necrosis factor; TSLP: Thymic Stromal Lymphophoietin.

**FIGURE 2:**
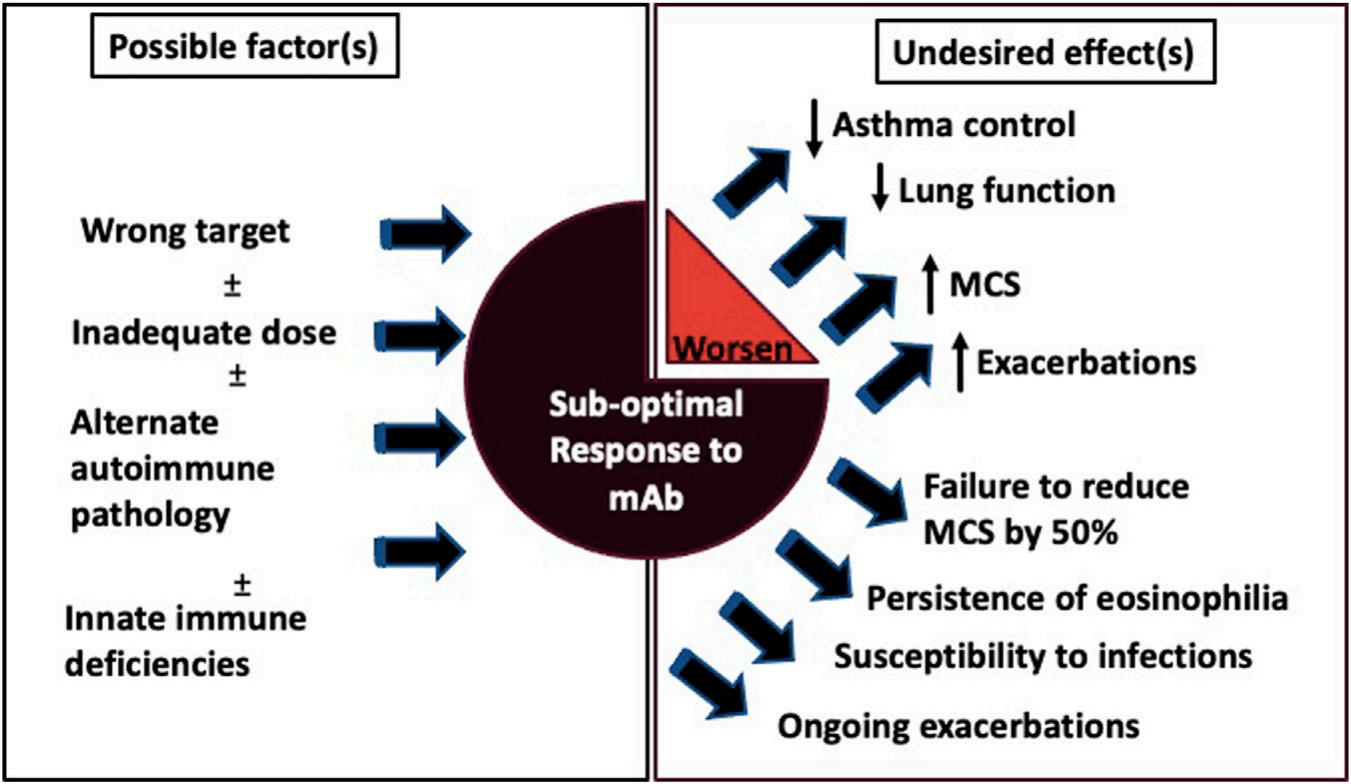
Factors affecting optimal biologic response and clinical ramifications. The schematic addresses the primary factors that alone or in combination can lead to sub-optimal treatment response to currently approved monoclonal antibodies (mAbs) in severe asthma. The sub-optimal responses to mAbs can result in undesired clinical manifestations *via*, persistence and worsening of asthma symptoms, exacerbations, infections and decline in lung functions. Abbreviations: mAb: Monoclonal anitbody; MCS: Maintenance corticosteroids.

The practice of precision medicine in asthma is far from optimal due to lack of complete understanding of the complex immunological nature of asthma. The severe asthma group is quite heterogeneous in nature and as such, a “one size fits all” approach cannot be used to manage these patients. Although many severe asthmatics have T2-high inflammation, the underlying mechanisms driving this inflammation may vary drastically across patients. This problem has been underscored by the high degree of variation in patient response to biologic therapy, where some patients respond dramatically and others either fail treatment or have suboptimal responses. Super-responders (SR) are defined as having improvement across three or more domains over a 12-month period including exacerbation elimination and improvement in asthma control (Upham et al., 2021). A better understanding is needed on how to identify these SR and determine what characteristics predispose them to a dramatic response to biologic therapy. Similarly, we need to better identify treatment failures and suboptimal responders to determine what underlying mechanisms contribute to this and how they differ from SR. In this section, we will review evidence behind current biologics and potential underlying mechanisms accountable for suboptimal response and treatment failure. The targeted pathways along with the key studies pertaining to these biologics are summarized in Figure 1 and Tables 1–7 respectively, while Figure 2 summarises the possible factors associated/contributing to poor therapeutic responses.

**TABLE 1:** Summary of anti-IgE targeted randomized clinical trials in severe asthma.

Anti-IgE						
Landmark study and year	Study type	Asthma severity	Asthma phenotype	Dosing, duration and route of administration	Clinical effect	Molecular effect
Hanania et al. (2013)	Phase 2	Severe uncontrolled asthma	Atopic	Dose: 0.016 mg/kg IgE (IU/ml)	-Reduced AAER	-Reduced FeNO
				Frequency: Q2W, Q4W	-Improved AQLQ, FEV_1_	
				Route: SC		
				Duration: 48 W		
INNOVATE, 2004 Busse et al. (2019)	Phase 2	Severe uncontrolled asthma	Atopic	Dose: 0.008–0.016 mg/kg IgE (IU/ml)	-Reduced AAER, ED visits	-N/A
				Frequency: Q2W, Q4W	-Improved morning PEF	
				Route: SC		
				Duration: 28 W		
Busse et al. (2001)	Phase 3	Severe uncontrolled asthma	Atopic	Dose: 0.008–0.016 mg/kg IgE (IU/ml)	-Reduced AAER, steroid dose	-Reduced serum IgE
				Frequency: Q4W	-Improved morning PEF, ACQ	
				Route: SC		
				Duration: 28 W		
Garcia et al. (2013)	Phase 3b	Severe uncontrolled asthma	Atopic	Dose: 200, 300 mg	-No change in ACQ or AAER	-FcεR1 decreased on basophils and DCs at 16 W
				Frequency: Q2W	-Increased FEV_1_	
				Route: SC		
				Duration: 16 W		
Djukanović et al. (2004)	Phase 3	Severe uncontrolled asthma	Atopic	Dose: 0.016 mg/kg IgE (IU/ml)	-No improvement in AHR	-Reduced SP, submucosal and epithelial eosinophils
				Frequency: Q4W		-Reduced FcεR1+ and IgE + cells, CD4/CD3/CD8 T cells, and IL-4+ cells in submucosa
				Route: SC		-Reduced serum IgE
				Duration: 16 W		
Chanez et al. (2010)	Phase 3	Severe uncontrolled asthma	Atopic	Dose: 0.016 mg/kg IgE (IU/ml)	-No change in AAER	-Reduced FcεR1 on basophils and DCs at 16 W
				Frequency: Q4W		
				Route: SC		
				Duration: 16 W		
de Llano et al. (2013)	Phase 3	Severe uncontrolled asthma	Atopic and non-atopic	Dose: 0.016 mg/kg IgE (IU/ml)	-Improved GETE scale and ACT score	-N/A
				Frequency: Q4W	-Increased FEV_1_	
				Route: SC	-No change in AAER	
				Duration: 24 M		
XCLUSIVE, 2011 Schumann et al. (2012)	Phase 3	Severe uncontrolled asthma	Atopic	Dose: 0.016 mg/kg IgE (IU/ml)	-Increased FEV_1_ at 16 W	-N/A
				Frequency: Q4W	-Improved ACQ at 16 W	
				Route: SC	-Reduced AAER	
				Duration: 6 M		
Holgate et al. (2005)	Phase 3	Severe uncontrolled asthma	Atopic	Dose: 0.016 mg/kg IgE (IU/ml)	-Reduced fluticasone dose	-N/A
				Frequency: Q4W		
				Route: SC		
				Duration: 16 W		
Barnes et al. (2013)	Retrospective study	Severe uncontrolled asthma	Atopic	Dose: 0.016 mg/kg IgE (IU/ml)	-Reduced steroid use/dose, hospitalizations and ED visits, and AAER	-N/A
				Frequency: Q4W	-Increased FEV_1_	
				Route: SC		
				Duration: 12 M		

AAER: annualized asthma exacerbation ratio; ACQ, asthma control questionnaire; AHR: airway hyperresponsiveness; ACT, asthma control test; AQLQ, asthma quality of life questionnaire; DC, dendritic cell; ED, emergency department; FeNO, fraction of expired nitric oxide; GETE, global evaluation of treatment effectiveness; IU, international units; M, months; NA, not applicable; Q, every; SC, subcutaneous; W, weeks.

**TABLE 2:** Summary of randomized clinical trials assessing mepolizumab in severe asthma.

Anti-IL-5: mepolizumab						
Landmark study and year	RCT phase	Asthma severity	Asthma phenotype	Dosing, duration and route of administration	Clinical effect	Molecular effect
Nair et al. (2009)	Phase 2	Severe uncontrolled asthma	≥300 cells/μl PB eosinophils in previous year or ≥150 cells/μl PB eosinophils at screening	Dose: 750 mg	-Reduced AAER and time-to-exacerbation	-No exacerbations associated with SP eosinophilia, instead there was SP neutrophilia
				Route: IV	-Reduction in prednisone dose	-Reduced SP and PB eosinophils
				Frequency: Q4W	-Improved FEV_1_ and ACQ	
				Duration: 26 W		
Haldar et al. (2009)		Severe uncontrolled asthma	≥3% sputum eosinophils in previous 2 years	Dose: 750 mg	-57% reduction in AAER at 50 W	-Reduced PB and SP eosinophils
				Route: IV	-Improved AQLQ score	-No change in FeNO or neutrophil count in SP
				Frequency: Q4W	-No change in FEV_1_ post-BD use or AHR	
				Duration: 52 W		
DREAM, 2012 Pavord et al. (2012); Ortega et al. (2016)	Phase 2	Severe uncontrolled asthma	≥0.3 × 10^9^ cells/L PB eosinophils or FeNO ≥50 ppb or SP eosinophils ≥3%	Dose: 75–750 mg	-48% reduction in exacerbations at 52 W	-Reduced PB and SP eosinophils
				Route: IV	-60% reduction in exacerbations requiring hospitalization or ED visits	
				Frequency: Q4W	-No difference in AQLQ, ACQ sores or FEV_1_	
				Duration: 52 W		
MENSA, 2014 Ortega et al. (2014); Ortega et al. (2016)	Phase 3	Severe uncontrolled asthma	≥300 cells/μl PB eosinophils in previous year or ≥150 cells/μl PB eosinophils at screening	Dose: 75, 100 mg	-53% reduction in AAER at 32 W	-Reduced PB eosinophils by 4 W
				Route: IV, SC	-61% reduction in ED visits or hospitalizations at 32 W	
				Frequency: Q4W	-Improved FEV_1_, PEF, SGRQ and ACQ (*p* < 0.05)	
				Duration: 32 W		
MUSCA, 2017 Chupp et al. (2017)	Phase 3b	Severe uncontrolled asthma	≥0.3 × 10^9^ cells/L PB eosinophils or FeNO ≥50 ppb or SP eosinophils ≥3%	Dose: 100 mg	-58% reduction in AAER at 24 W	-N/A
				Route: IV	-68% reduction in hospitalizations and ED visits at 24 W	
				Frequency: Q4W	-Improvement in SQRQ score at 4 W	
				Duration: 52 W	-Improved pre-BD FEV_1_ at 24 W	
SIRIUS, 2017 Bel et al. (2014)	Phase 3	Severe uncontrolled asthma	≥300 cells/μl PB eosinophils in previous year or ≥150 cells/μl PB eosinophils at screening	Dose: 100 mg	-32% reduction in AAER 24 W	-N/A
				Route: IV	-50% reduction in OCS dose at 24 W	
				Frequency: Q4W	-Improved ACQ and SQRQ at 24 W	
				Duration: 52 W	- No change in FEV_1_ at baseline or post-BD	
COSMOS, 2016 Lugogo et al. (2016)	Phase 3	Severe uncontrolled asthma	≥300 cells/μl PB eosinophils in previous year or ≥150 cells/μl PB eosinophils at screening	Dose: 100 mg	-Maintained reduced exacerbation rates and OCS dosing	-N/A
				Route: SC		
				Frequency: Q4W		
				Duration: 52 W		
COLUMBIA, 2019 Khatri et al. (2019)	Phase 3	Severe uncontrolled asthma	≥300 cells/uL PB eosinophils in previous year or ≥150 cells/μl PB eosinophils at screening	Dose: 100 mg	-61% reduction in AAER	-N/A
				Route: SC	-Improved ACQ-5 at 24 W	
				Frequency: Q4W	-Improved pre-BD FEV_1_ at 24 W	
				Duration: 52 W		
COSMEX, 2019 Khurana et al. (2019)	Phase 3b	Severe uncontrolled asthma	≥300 cells/μl PB eosinophils in previous year or ≥150 cells/μl PB eosinophils at screening	Dose: 100 mg	-Maintained reduced AAER and daily OCS use	-Reduced PB eosinophils
				Route: SC	-Improved FEV_1_ and ACQ-5	
				Frequency: Q4W		
				Duration: 172 W		
OSMO, 2019 Chapman et al. (2019)	Phase 4	Severe uncontrolled asthma	≥300 cells/μl PB eosinophils in previous year or ≥150 cells/μl PB eosinophils at screening	Dose: 100 mg	-64% reduction in AAER at 32 W	-Reduced blood eosinophils, ECP, EDN at 32 W
				Route: SC	-69% reduction in hospitalizations and ED visits at 32 W	
				Frequency: Q4W	-Improved SGRQ and pre-BD FEV_1_ at 32 W	
				Duration: 32 W		
Bagnasco et al. (2018)	Prospective cohort study	Severe uncontrolled asthma	≥300 cells/μl PB eosinophils in previous year or ≥150 cells/μl PB eosinophils at screening	12 M post-initiation of mepolizumab	-Reduction in OCS-dependence and exacerbation rate	-N/A
REALITI-A, 2020 Harrison et al. (2020)	Prospective cohort study	Severe uncontrolled asthma	<300 cells/μl or ≥300 cells/μl PB eosinophils	12 M post-initiation of mepolizumab	-Reduced AAER, hospitalizations and ED visits	-Reduced PB eosinophils
					-Reduced OCS maintenance dose	
Pelaia et al. (2018a); Pelaia et al. (2018b)	Single-centered observational study	Severe uncontrolled asthma	≥300 cells/μl PB eosinophils in previous year or ≥150 cells/μl PB eosinophils at screening	Dose: 100 mg	-Increased ACT score after 24 W	-Reduced PB eosinophils at 24 W
				Route: SC	-Improved FEV_1_ and FEV_1_/FVC after 24 W	
				Frequency: Q4W	-Reduced exacerbation frequency	
				Duration: 24 W	-Decreased prednisone dose	

**TABLE 3:** Summary of randomized clinical trials assessing reslizumab in severe asthma.

Anti-IL-5: reslizumab						
Landmark study and year	Study format	Asthma severity	Asthma phenotype	Dosing, duration and route of administration	Clinical effect	Molecular effect
Castro et al. (2011)	Phase 2	Severe uncontrolled asthma	≥400 cells/μl PB eosinophils	Dose: 3 mg/kg	-Improved FEV_1_ but no change in ACQ or AAER	-Reduced SP eosinophils
				Route: IV		
				Frequency: Q4W		
				Duration: 15 W		
Castro et al. (2015)	Phase 3	Severe uncontrolled asthma	≥400 cells/μl PB eosinophils	Dose: 3 mg/kg	-Reduced AAER	-N/A
				Route: IV		
				Frequency: Q4W		
				Duration: 52 W		
Bjermer et al. (2016)	Phase 3	Severe uncontrolled asthma	≥400 cells/μl PB eosinophils	Dose: 3 mg/kg	-Improved ACQ, AQLQ, FEV_1_ and FVC	-N/A
				Route: IV		
				Frequency: Q4W		
				Duration: 16 W		
Corren et al. (2016)	Phase 3	Severe uncontrolled asthma	≥400 or <400 cells/μl PB eosinophils	Dose: 3 mg/kg	-No change in mean FEV_1_, except in subgroup analysis with eosinophilia	-Reduced PB eosinophils
				Route: IV		
				Frequency: Q4W		
				Duration: 16 W		
Murphy et al. (2017)	Phase 3	Severe uncontrolled asthma	≥400 cells/μl PB eosinophils	Dose: 3 mg/kg	-Improved ACQ, AQLQ, FEV_1_ and FVC	-Reduced PB eosinophils
				Route: IV		
				Frequency: Q4W		
				Duration: 24 M		
Brusselle et al. (2017)	Phase 3	Severe uncontrolled asthma	≥400 cells/μl PB eosinophils	Dose: 3 mg/kg	-Reduced AAER over 52W, and exacerbations requiring hospitalization/ED visits	-N/A
				Route: IV	-Improved ACQ and AQLQ for late onset patients	
				Frequency: Q4W		
				Duration: 52 W		
Weinstein et al. (2019)	Phase 3	Severe uncontrolled asthma with CRS	≥400 cells/μl PB eosinophils	Dose: 3 mg/kg	-Reduced AAER	-N/A
				Route: IV	-Improved FEV_1_	
				Frequency: Q4W		
				Duration: 52 W		
Bernstein et al. (2020)	Phase 3	Severe uncontrolled asthma	≥300 cells/μl PB eosinophils	Dose: 110 mg	-No difference in AAER, except in those with PB eosinophils ≥400	-N/A
				Route: SC	-No difference in steroid dosing	
				Frequency: Q4W		
				Duration: 52 W		
Mukherjee et al. (2018b)	Placebo-Controlled Sequential Trial	Severe uncontrolled asthma previously on 1Y of mepolizumab	≥3% SP and ≥300 cells/μl PB eosinophils	Dose: 3 mg/kg	-Improved FEV_1_ and ACQ	-Reduced SP and PB eosinophils, SP EPX, anti-EPX, and ANA
				Route: IV		-Reduced PB HPC, EoP, and SP CD4^+^ T cells, no change in ILC2 in PB or SP
				Frequency: Q4W		
				Duration: 12 W		
Ibrahim et al. (2019)	Prospective Cohort	Severe uncontrolled asthma	≥400 cells/μl PB eosinophils	Dose: 3 mg/kg	-Improved ACQ at baseline and up to 2Y	-Reduced PB eosinophils
				Route: IV	-Reduced OCS dose and/or use at 1Y	
				Frequency: Q4W	-Reduced AAER at 1Y	
				Duration: 2 Y		
Pérez de Llano et al. (2019)	Open-label Prospective Study	Severe uncontrolled asthma who failed omalizumab	≥400 cells/μl PB eosinophils	Dose: 3 mg/kg	-Improved ACT score at 4, 12 W	-Reduced PB eosinophils and FeNO at 24 W
				Route: IV	-Improved ACQ score at 12, 24 W	
				Frequency: Q4W	-60% of patients controlled at 24 W	
				Duration: 24 W		

**TABLE 4:** Summary of randomized clinical trials assessing benralizumab in severe asthma.

Anti-IL-5R alpha: benralizumab						
Landmark study and year	Study format	Asthma severity	Asthma phenotype	Dosing, duration and route of administration	Clinical effect	Molecular effect
Nowak et al. (2015)	Phase 2	Severe uncontrolled asthma	<450 cells/μl or ≥450 cells/μl PB eosinophils	Dose: 0.3 mg/kg or 1 mg/kg	-49% reduction in AAER	-Reduced PB eosinophils up to 12 W
				Route: SC	-60% reduction in hospitalization	-Reduced ECP and EDN
				Frequency: Once	-No change in FEV_1_, ACQ, AQLQ	
				Duration: 24 W		
CALIMA, 2016 FitzGerald et al. (2016)	Phase 3	Severe uncontrolled asthma	<300 cells/μl or ≥300 cells/μl PB eosinophils	Dose: 30 mg	-28% reduction in AAER at 56 W	-Reduced PB eosinophils
				Route: SC	-Improved ACQ, AQLQ, pre-BD FEV_1_	
				Frequency: Q4W, Q8W		
				Duration: 56 W		
SIROCCO, 2016 Bleecker et al. (2016)	Phase 3	Severe uncontrolled asthma	<300 cells/μl or ≥300 cells/μl PB eosinophils	Dose: 30 mg	-Reduced AAER, regardless of eosinophils at 48 W	-Reduced PB eosinophils by 4 W
				Route: SC		
				Frequency: Q4W, Q8W	-Improved FEV_1_, ACQ-6, AQLQ at 48 W	
				Duration: 48 W		
ZONDA, 2017 Nair et al. (2017)	Phase 3	Severe uncontrolled asthma	≥150 cells/μl PB eosinophils	Dose: 30 mg	-75% reduction in OCS dose	-N/A
				Route: SC	-Improved AAER, ACQ-6, AQLQ	
				Frequency: Q4W, Q8W	-No effect on FEV_1_	
				Duration: 28 W		
BISE, 2017 Ferguson et al. (2017)	Phase 3	Mild-moderate persistent asthma	<300 cells/μl or ≥300 cells/μl PB eosinophils	Dose: 30 mg	-Increased pre-BD FEV_1_ at 12 W	-N/A
				Route: SC		
				Frequency: Q4W		
				Duration: 12 W		
Chupp et al. (2019)	Phase 3	Severe uncontrolled asthma	≥300 cells/μl PB eosinophils	Dose: 30 mg	-Improvement in morning PEF from baseline within 2 W	-N/A
				Route: SC		
				Frequency: Q4W, Q8W		
				Duration: 28–56 W		
BORA, 2019 Busse et al. (2019)	Phase 3	Severe uncontrolled asthma	<300 cells/μl or ≥300 cells/μl PB eosinophils	Dose: 30 mg	-72% of patients with eosinophilia did not have exacerbation	-N/A
				Route: SC	-Maintained improvement in FEV_1_ and ACQ, AQLQ	
				Frequency: Q4W Q8W		
				Duration: 56 W		
Gauvreau et al. (2021)	RCT	Mild asthma	Atopic	Dose: 30 mg	-N/A	-Reduced SP eosinophils at 7 h post-allergen challenge
				Route: SC		-Reduced PB, BM and SP before and 24 h post-allergen challenge
				Frequency: Q4W		-Incomplete depletion of basophils in PB and BM pre- and post-24 h allergen in challenge, no effect on SP basophils
				Duration: 12 W		
Sehmi et al. (2018)	RCT	Severe uncontrolled asthma	≥3% sputum eosinophils	Dose: 30 mg	-N/A	-Reduced BP and SP eosinophils
				Route: SC		-Reduced PB EoP
				Frequency: Q4W, Q8W		-Reduced IL-5-stimulated Eo/B CFU
				Duration: 28 W		
J-BEST, 2019 Izumo et al. (2020)	Prospective	Severe uncontrolled asthma	≥300 cells/μl PB eosinophils	Dose: 30 mg	-Improved FEV_1_, ACT, AQLQ	-Decreased PB eosinophils and basophils, but no change in FeNO or serum total IgE
				Route: SC		
				Frequency: Q4W, Q8W		
				Duration: 4–12 W		
Kavanagh et al. (2021)	Prospective	Severe uncontrolled asthma	≥400 cells/μl PB eosinophils	Dose: 30 mg	-Reduced AAER	-Reduced PB eosinophils
				Route: SC	-Improved FEV_1_, ACQ, AQLQ	-No change in FeNO
				Frequency: Q4W Q8W		
				Duration: 48 W		
PONENTE, 2019 (ongoing) Menzies-Gow et al. (2019)	Phase 3b	Severe uncontrolled asthma	≥150 cells/μl PB eosinophils at enrollment or ≥300 cells/μl PB eosinophils in last 12 M	Dose: 30 mg	Pending	Pending
				Route: SC		
				Frequency: Q4W Q8W		
				Duration: 32 W		

**TABLE 5:** Summary of randomized clinical trials assessing IL-4/13 in severe asthma.

Anti-IL-4/13						
Landmark study and year	RCT phase	Asthma severity	Inflammatory profile	Dosing, duration and route of administration	Clinical effect	Molecular effect
Hodsman et al. (2013)	Phase 1	Mild asthma	Independent of PB eosinophils	Biologic: GSK679586	-N/A	-Increased serum IL-13
				Dosing: 0.005–10 mg/kg		-Reduced FeNO at 2W and 8 W
				Frequency: Q4W		
				Route: IV		
				Duration: 28 W		
De Boever et al. (2014)	Phase 2	Severe uncontrolled asthma	≥140 cells ul PB eosinophils	Biologic: GSK679586	-No change in ACQ score, FEV_1_, AAER	-No difference in serum IL-13 or IgE
				Dosing: 10 mg/kg Frequency: Q4W		-No difference in PB eosinophils
				Route: IV		
				Duration: 24 W		
Piper et al. (2013)	Phase 2a	Moderate-severe uncontrolled asthma	Independent of PB eosinophils	Biologic: Tralokinumab	-No change in ACQ, pre-BD FEV_1_, FVC, PEF, AAER.	N/A
				Dosing: 150–600 mg		
				Frequency: Q2W		
				Route: SC		
				Duration: 24 W		
Russell et al. (2018)	Phase 2	Moderate-severe uncontrolled asthma	Independent of PB eosinophils	Biologic: Tralokinumab	-N/A	-No change in bronchial eosinophils at 12 W
				Dosing: 300 mg		- No change in PB or SP eosinophils or serum IgE
				Frequency: Q2W		
				Route: SC		
				Duration: 12 W		
STRATOS I, II, 2018; Panettieri et al. (2018)	Phase 3	Severe uncontrolled asthma	≥37 ppb FeNO or <37 ppb	Biologic: Tralokinumab	-Reduced AAER at 2 W in FeNO-high patients	-N/A
				Dosing: 300 mg		
				Frequency: Q2W, Q4W		
				Route: SC		
				Duration: 52 W		
Corren et al. (2011)	Phase 2	Severe uncontrolled asthma	Periostin ≥50 or <50 ng/ml	Biologic: Lebrikizumab	-60% reduction in exacerbation at 24 W	-19% reduction in FeNO at 12 W
				Dose: 250 mg	-Improved FEV_1_, at 12 W	-Decreased CCL13, CCL17, total IgE levels at 24 W
				Frequency: Q4W	-No change in ACQ	
				Route: SC		
				Duration: 24 W		
Noonan et al. (2013)	Phase 2	Mild asthma	Periostin ≥50 or <50 ng/ml	Biologic: Lebrikizumab	-No change in FEV_1_, pre-PB PEF, AQLQ	-N/A
				Dose: 125–500 mg		
				Frequency: Q4W		
				Route: SC		
				Duration: 12 W		
LUTE, VERSE, 2015 Hanania et al. (2015)	Phase 3	Severe uncontrolled asthma	Periostin ≥50 or <50 ng/ml	Biologic: Lebrikizumab	-60% reduction in exacerbation in periostin-high patients	- Reduction in PB eosinophils and FeNO
				Dose: 37.5–250 mg	-No dose response for exacerbation	
				Frequency: Q4W	-Improved FEV_1_ at 12 W	
				Route: SC		
				Duration: 52 W		
LAVOLTA I, II, 2016; Hanania et al. (2016)	Phase 2	Severe uncontrolled asthma	≥300 cells μl PB eosinophils or periostin ≥50 ng/ml	Biologic: Lebrikizumab	-70% reduction in exacerbation in periostin- high patients	-N/A
				Dose: 37.5–125 mg		
				Frequency: Q4W		
				Route: SC		
				Duration: 52 W		
STRETTO, 2018; Korenblat et al. (2018)	Phase 3	Mild-moderate asthma	≥300 cells μl PB eosinophils or periostin ≥50 ng/ml	Biologic: Lebrikizumab	-No change in FEV_1_, pre-PB PEF, AQLQ	-N/A
				Dose: 125 mg		
				Frequency: Q4W		
				Route: SC		
				Duration: 12 W		
Wenzel et al. (2007)	Phase 2a	Atopic asthma	Independent of PB eosinophils	Biologic: Pitrakinra	-No change in FEV_1_ for SC trial but reduction with inhaled	- Decreased FeNO with inhaled group
				Dose: 25, 60 mg		- No change in SP or PB eosinophils
				Frequency: OD, BID		- No change in serum IgE
				Route: SC, Inhaled		
				Duration: 12 W		
Otulana et al. (2011)	Phase 2b	Moderate-severe uncontrolled asthma	Independent of blood eosinophils or atopic status	Biologic: Pitrakinra	-Reduced exacerbation in eosinophilic group	-N/A
				Dose: 1–10 mg BID	-Improvement in symptom scores and spirometry	
				Route: Inhaled		
				Duration: 12 W		
Corren et al. (2010)	Phase 2	Moderate-severe asthma	Independent of blood eosinophils or atopic status	Biologic: AMG 317	-No improvement in ACQ	- No change in serum IgE
				Dose: 75–300 mg	-No decrease in exacerbation	- No change in sputum eosinophils, FeNO
				Frequency: Q4W		
				Route: SC		
				Duration: 12 W		
Wenzel et al. (2013)	Phase 2	Moderate-severe asthma	≥300 cells μl PB eosinophils or SP eosinophils ≥3%	Biologic: Dupilumab	-87% reduction in exacerbation at 12 W	- Reduced FeNO from 4 to 12 W
				Dose: 300 mg	-Increase in FEV_1_ predicted from 2 to 12 W	- Decrease in serum TARC, eotaxin-3 or IgE
				Frequency: Q1W	-Improved ACQ at 3 W	- No change in PB or SP eosinophils
				Route: SC		
				Duration: 12 W		
Wenzel et al. (2016)	Phase 2b	Severe uncontrolled asthma	<300 cells/ul or ≥300 cells/μl PB eosinophils	Biologic: Dupilumab	-Increased FEV_1_ in those with PB ≥ 300 cells/uL eosinophils	-Reduced FeNO at 24 W
				Dose: 200, 300 mg	-Reduced AAER	
				Frequency: Q2-4W	-Improved ACQ, regardless of eosinophils	
				Route: SC		
				Duration: 24 W		
LIBERTY ASTHMA QUEST, 2018; Castro et al. (2018)	Phase 3	Severe uncontrolled asthma	<300 cells/μl or ≥300 cells/μl PB eosinophils	Biologic: Dupilumab	-Reduced AAER	-Reduced FeNO, serum IgE, periostin, eotaxin-3, TARC at 52 W
				Dose: 200, 300 mg	-Increased FEV_1_ at 12 W	-Transient increased PB eosinophils with increased ECP
				Frequency: Q2W	-Improved ACQ, AQLQ scores	
				Route: SC		
				Duration: 52 W		
LIBERTY ASTHMA QUEST, 2018; Castro et al. (2018)	Phase 3	Severe uncontrolled asthma	<300 cells/μl or ≥300 cells/μl PB eosinophils	Biologic: Dupilumab	-Reduced OCS dose	-Reduced FeNO
				Dose: 300 mg	-Reduced rate of severe asthma exacerbations and AAER	-Transient increased PB eosinophils
				Frequency: Q2W	-Increased FEV_1_	
				Route: SC	-Improved ACQ	
				Duration: 24 W		
LIBERTY ASTHMA VENTURE, 2020; Rabe et al. (2020)	Phase 3	Severe uncontrolled asthma	<300 cells/μl or ≥300 cells/μl PB eosinophils	Biologic: Dupilumab	-Improved pre-BD FEV_1_, FVC, FEV_1_/FVC	-N/A
				Dose: 300 mg	- Reduced AAER	
				Frequency: Q2W		
				Route: SC		
				Duration: 24 W		
Maspero et al. (2020)	Phase 3	Moderate-severe uncontrolled asthma and CRS	≥150 cells/μl or ≥300 cells/μl PB eosinophils	Biologic: Dupilumab	-Increased pre- and post-BD FEV_1_ in CRS and non-CRS groups	-Decrease in FeNO, serum IgE and TARC in CRS and non-CRS groups
				Dose: 200, 300 mg	-Improved ACQ, AQLQ, SNOTT-22 scores in CRS and non-CSR groups	-No change in PB eosinophils in non-CRS group, but mild elevation in CRS group
				Frequency: Q2W		
				Route: SC		
				Duration: 24 W		

**TABLE 6:** Summary of randomized clinical trials assessing tezepelumab in severe asthma.

Anti-TSLP: tezepelumab						
Landmark study and year	RCT phase	Asthma severity	Biomarker	Dosing, duration and route of administration	Clinical effect	Molecular effect
Gauvreau et al. (2014)	Phase 1b	Mild allergic asthma	Atopic, independent of PB Eosinophils	Dosing: 700 mg Q4W	34% improvement in FEV_1_ on day 84 (*p* = 0.02) compared to placebo	- PB eosinophils declined post-dosing and reached normal levels by 4 W
				Route: SQ		- SP eosinophils reached normal levels by 6 weeks
				Duration: 12 W		- FeNO levels improved 1 W post-first dose
						- No effect on total IgE levels
PATHWAY, 2017, 2021, 2021; Corren et al. (2017); Corren et al. (2021a); Corren et al. (2021b)	Phase 2b	Severe uncontrolled asthma	PB Eosinophils ≥250 or <250 cells/μl	Dosing	- 62–71% reduction in exacerbation irrespective of phenotype, across all seasons	- Decrease in PB eosinophils in all tezepelumab groups at 4 W onwards
				700 mg Q4W	- Reduced asthma-exacerbation related hospitalizations	- Decrease in total serum IgE all tezepelumab groups
				210 mg Q4W	- FEV_1_ 120–150 ml improvement vs. placebo (*p* = 0.002–0.015)	
				280 mg Q2W	-Significant improvement in ACQ and AQLQ scores in higher-dose intervention arms	
				Route: SQ		
				Duration: 52 W		
NAVIGATOR, 2021; Menzies-Gow et al. (2021)	Phase 3	Severe uncontrolled asthma	<300 cells/μl or ≥300 cells μl PB eosinophils	Dosing	- Reduced AAER by 44–59% vs. placebo, irrespective of phenotype	- Decrease in PB eosinophils and FeNO levels at 2 W onwards vs. placebo
				210 mg Q4W	- FEV_1_ 230 ml improvement vs. placebo (*p* < 0.0001)	- Serum IgE levels reduced over 5 W vs. placebo
				Route: SQ	- Significant improvement in ACQ and AQLQ	
				Duration: 52 W		
UPSTREAM (2021); Sverrild et al. (2021)	Phase 2	Severe uncontrolled asthma	Independent of blood eosinophils or atopic status	Dosing: 700 mg Q4W	- Mean change in PD_15_ significantly reduced	- Airway tissue and BAL eosinophils decreased by 74 and 75%, respectively (*p* = 0.004, *p* = 0.01)
				Route: SQ	- Non-significant improvement in ACQ	- No significant change in tissue mast cells
				Duration: 12 W		- Subepithelial neutrophils increased by 51% with tezepelumab vs. 33% in placebo (non-significant)
CASCADE, 2020 (Ongoing), 2021 Diver et al. (2021)	Phase 2	Moderate-severe uncontrolled asthma	<300 cells/μl or ≥300 cells μl PB eosinophils	Dosing: 210 mg Q4W	-Reduced AHR to mannitol vs. placebo	- Decreased submucosal eosinophils vs. placebo, regardless of baseline PB eosinophils
				Route: SQ		- No difference in CD3^+^ T cells or CD4^+^ T cells, mast cells
				Duration: 28 W		- No difference in reticular basement membrane thickness and epithelial integrity
SOURCE, 2020 (Ongoing) Wechsler et al. (2020b)	Phase 3	Severe uncontrolled asthma	<300 cells/μl or ≥300 cells μl PB eosinophils	Dosing: 210 mg Q4W	Pending	Pending
				Route: SQ		
				Duration: 48 W		
DESTINATION, 2020 (Ongoing) Menzies-Gow et al. (2020)	Phase 3	Severe Uncontrolled Asthma	<300 cells/μl or ≥300 cells μl PB eosinophils	Dosing: 210 mg Q4W	Pending	Pending
				Route: SQ		
				Duration: 36 W		

**TABLE 7:** Summary of IL-33/ST2-targeted therapy in severe asthma.

Anti-IL-33/ST2						
Landmark study and year	RCT phase	Disease model	Disease phenotype	Dosing, duration and route of administration	Clinical effect	Molecular effect
Chen et al. (2019)	Phase 2a	Atopic dermatitis	Atopic	Biologic: Etokimab	-83% achieved EASI50 and 33% EASI75	- Reduction in PB eosinophils at day 29
				Dosing: 300 mg		- Reduction in skin neutrophil infiltration post-HDM skin challenge
				Route: IV		- Inhibited neutrophil migration to skin
Chinthrajah et al. (2019)	Phase 2a	Peanut allergy	Atopic	Biologic: Etokimab	- Significant desensitization to peanuts	- Reduction in cytokine levels (IL-4, IL-5, IL-9, IL-13), and ST2 levels in CD4^+^ T cells in PB
				Dosing: 300 mg		- Reduction in IgE at day 15
				Route: IV		
				Duration: 6 W		
Wechsler et al. (2021)	Phase	Moderate-severe asthma	<200 cells/μl or ≥200 cells μl PB eosinophils	Biologic: Itepekimab	- Reduction in loss of asthma control (22%)	- Reduction in mean blood eosinophil count, FeNO, serum total IgE, periostin, plasma eotaxin-3, and serum pulmonary and activation-regulated chemokine (PARC)
				Dosing: 300 mg	- No improvement in FEV1	
				Route: SQ	- Improvement in AQLQ and ACQ	
				Duration: 12 W		
NCT03207243, 2020 (Ongoing)	Phase 2a	Moderate-severe asthma	Independent of blood eosinophils or atopic status	Biologic: GSK3772847	Pending	Pending
				Dosing: 10 mg/kg Q4W		
				Route: IV		
				Duration: 28 W		
NCT02918019, 2020 (Ongoing)	Phase 2b	Severe uncontrolled asthma	Independent of blood eosinophils or atopic status	Biologic: MSTT1041A	Pending	Pending
				Dosing: 210 mg		
				Q4W		
				Route: SQ		
				Duration: 50 W		

### 3.1 IgE Targeted Therapy

IgE is the primary immunoglobulin involved in T2-high inflammation. Omalizumab binds to the third constant region of IgE and prevents free IgE from interacting with high and low-affinity Fc**ε**R1 receptors (Fahy et al., 1997). As a result of this binding, free serum IgE levels decrease, as well as the overall IgE receptor density on mast cells and basophils. Numerous randomized clinical trials (RCTs) and real-life studies have shown that treatment of asthmatics with omalizumab results in a dose-dependent reduction in free IgE in serum, improvement in lung function, and modest reduction in exacerbation rates, as well as emergency visits and hospitalizations (Corne et al., 1997; Hanania et al., 2011; Rodrigo et al., 2011; Normansell et al., 2014). RCTs have also shown improvement in symptom control, quality of life, and reduced oral corticosteroid (OCS) use (Table 1) (Humbert et al., 2005; Rodrigo et al., 2011; Normansell et al., 2014; Pelaia et al., 2018a). In terms of molecular findings, omalizumab reduces both eosinophil and basophil infiltration within the airways (Rodrigo et al., 2011; Normansell et al., 2014; Pelaia et al., 2018a). Of note, a large retrospective analysis of 25 RCTs demonstrated greater reduction in asthma exacerbation patients who specifically had high blood eosinophilia and fractional exhaled nitric oxide (FeNO) levels, which was suggestive of eosinophilic inflammation (Hanania et al., 2013). Based on these findings, it seemed justified to prescribe anti-IgE biologics to severe asthmatics with evidence of atopy. As promising as the aforementioned studies are, they were primarily based on mild-moderate asthmatics, and more studies are needed to determine efficacy of these agents in severe corticosteroid-dependent asthma.

#### 3.1.1 Possible Reasons for Suboptimal Responses With Anti-IgE

Agents that target IgE-dependent mechanisms have been shown to be efficacious in mild to moderate asthma, however, this pathway may not be the major driver of eosinophilic inflammation in severe asthma. Thus, although anti-IgE agents may sufficiently suppress IgE-dependent mechanisms, the IgE-independent pathways are still active within the airways, continuing to drive Th2 inflammation. This is, in part, supported by studies showing that omalizumab treatment in severe asthma does not reduce sputum eosinophils, (Mukherjee et al., 2019) and that pediatric patients with severe asthma are low responders to omalizumab (Garcia et al., 2013). Chapman et al. (2019) assessed the efficacy of using mepolizumab to treat severe eosinophilic asthmatics who were inadequately controlled on omalizumab. Interestingly, subgroup analysis demonstrated no additional benefit when both biologics were in the system, nor was there a decline seen in the benefit of omalizumab as it washed out. However, the patients who showed the most improvement in asthma control were those with eosinophilia (≥150 cells/μl blood eosinophils). These findings suggest that singular targeting of IgE-dependent mechanisms may not be effective in all inflammatory subtypes of severe asthma and that perhaps targeting IgE is more beneficial in patients with underlying atopic status. In addition, there have been reports of IgG autoantibodies generated against IgE in allergic asthma and the formation of IgE-IgG heterocomplexes in autoimmune conditions that trigger innate immune cells (Chan et al., 2014; Henault et al., 2016). Thus, the presence of autoantibodies and immune complexes in allergic airways may impede the action of anti-IgE mAb and inadvertently induce a continued need for OCS. More studies are needed to determine what particular inflammatory profiles of severe asthma would benefit most from IgE blockade with omalizumab and if its combination with agents that target IgE-independent mechanisms would provide a synergistic effect.

### 3.2 IL-5 Targeted Therapy

Given the role of IL-5 in driving eosinophilic inflammation, it was proposed that blockade of this cytokine may attenuate T2-high inflammation. There have been three agents developed so far that target IL-5. Mepolizumab and reslizumab bind to IL-5, thereby preventing this cytokine from promoting eosinophil activation. Benralizumab, alternatively blocks the IL-5Rα, resulting in near complete depletion of peripheral eosinophils through antibody-dependent cell-mediated cytotoxicity (ADCC) involving NK cells.

The story of mepolizumab provides a valuable lesson for individualized asthma management. Leckie et al. first showed in 2000 that despite mepolizumab resulting in attenuation of blood and sputum eosinophils, there was a lack of translation into meaningful clinical outcomes in patients with asthma (Leckie et al., 2000). Large RCTs later followed, which also failed to show clinical efficacy, and as a result the development of anti-IL-5, or indeed any anti-eosinophil therapy, was tabled for many years (Flood-Page et al., 2003). In fact, the overall importance of IL-5 and eosinophils in asthma was brought into question. It should be noted that these initial trials did not select appropriate patient populations based on eosinophilia and a T2-high profile, but rather selected a heterogeneous pool of asthmatic patients with a variety of immunological profiles. Hence, it is not surprising that there was a lack of clinical response to anti-IL-5 treatment in these early studies.

This led to a pivotal change in studies examining anti-IL-5 therapy through the specific targeting of T2-high patients. Using patient selection criteria for the T2-high profile, Haldar et al. reported that mepolizumab treatment in severe asthmatics that specifically exhibited eosinophilia (≥3% sputum eosinophils in last 12 months), not only reduced blood and sputum eosinophils but also resulted in 43% fewer exacerbations (Haldar et al., 2009). Further, Nair et al. showed that mepolizumab administration to severe prednisone-dependent eosinophilic asthmatics resulted in OCS tapering, where patients had an 83% reduction of their maximum prednisone dose versus 47% with placebo. They also reported fewer exacerbations, improved asthma control, and increased FEV_1_ with an associated decrease in sputum and blood eosinophils (Nair et al., 2009). Multiple other RCTs and real-world investigations have shown that mepolizumab has a corticosteroid-sparing effect in this population, with reductions in annual asthma exacerbation rates (AAER) by 39–52%, improvement in lung function, and asthma symptom scores, as well as improvement in overall health-related quality of life (Table 2) (Pavord et al., 2012; Bel et al., 2014; Chupp et al., 2017; Pelaia et al., 2018b; Harrison et al., 2020).

Three important observations should be noted from the mepolizumab data. Firstly, the efficacy of mepolizumab is based on patients having baseline eosinophilia, and as such, we must identify individuals that will benefit from this treatment in the first place. Peripheral blood eosinophils were initially chosen as a biomarker in the mepolizumab studies for a few reasons. Peripheral eosinophils have been identified through cluster analyses to predict responsiveness to mepolizumab (Ortega et al., 2016). Indeed, it is one of the most simple and practical ways to identify Th2 inflammation and as such, patients that may benefit from anti-IL-5 therapy. The caveat to this is that peripheral eosinophils can be highly variable and a single measurement of peripheral blood eosinophils may not reflect the average level of cells throughout an extended period of time. Thus, the use of peripheral blood eosinophils to guide therapy to anti-IL-5 biologics may not be as ideal as sputum eosinophils. Unfortunately, there is a lack of global availability of sputum labs making it difficult to use sputum eosinophils as a universal biomarker. However, if available, sputum counts can be reproducible and reliable if the proper processing technique is done. We propose that peripheral eosinophilia may be helpful to predict response to anti-IL-5 agents but has a limited role in monitoring response to treatment. As previously reported, in 250 patients with baseline blood eosinophilia (≥400 cells/μl) treated with either mepolizumab or reslizumab for at least 4 months, there was an overall suboptimal response in 43% (Mukherjee et al., 2018b). Of the 129 patients in whom paired blood and sputum eosinophils were available 4 months post-treatment, there were 65 suboptimal responders, 78% of who had sputum eosinophils ≥3%. Only seven of these patients had blood eosinophils ≥400 cells/μl. As such, there is a discordance between the two compartments, which may be, in part, due to *in situ* eosinophilopoiesis. As such, the use of sputum eosinophils to monitor response to treatment may be more reliable than peripheral eosinophils. Secondly, despite multiple studies showing clinical benefit, the effect of mepolizumab was ultimately incomplete, with ∼50% reduction in exacerbation rates despite ablation of peripheral eosinophilia. This begs the question as to whether peripheral blood eosinophils should be the only biomarker to determine whether patients would benefit from this biologic. Lastly, and most importantly, we need to re-examine the spotlight on eosinophils and their overall importance in T2-high inflammation. Aside from eosinophils, there are cytokines and effector cells, which may be equally, if not more important than eosinophils, and as such, should be targeted.

Reslizumab was the second anti-IL-5 biologic to be brought to market. In contrast to mepolizumab, it is administered intravenously with weight-based dosing. A phase 2 RCT showed that reslizumab reduced both sputum and blood eosinophils, with an associated improvement in FEV_1_ (Kips et al., 2003). Subsequent RCTs in severe asthmatics with blood eosinophils ≥400 cells/μl, have shown that reslizumab reduces AAER by 50–60%, improves symptom scores and lung function, and reduces blood eosinophils (Table 3) (Castro et al., 2011; Castro et al., 2015; Bjermer et al., 2016; Corren et al., 2016; Brusselle et al., 2017; Máspero, 2017; Murphy et al., 2017; Bernstein et al., 2020). Finally, post-hoc analysis suggested that patients who do not respond to the fixed-dose regimen of mepolizumab may benefit from reslizumab as an alternative (Mukherjee et al., 2018b). Similar to mepolizumab, reslizumab seems to have the most evidence for clinical efficacy in those with peripheral eosinophilia, however, the reduction in exacerbation rates remains incomplete.

Benralizumab, an antibody to the alpha subunit of the IL-5 receptor, was the third IL-5 pathway-targeting biologic that came to fruition. Phase 3 RCTs have shown that severe eosinophilic asthmatics (≥150 cells/μl blood eosinophils in last 12 months) treated with benralizumab 30 mg SC q4 weeks, were able to reduce their prednisone dosing, exhibited reduced AAER by 28–55%, and also showed improved lung function and symptom scores. From a cellular and molecular standpoint, benralizumab reduced peripheral and sputum eosinophils, along with diminished eosinophil products, including ECP and EDN (Nowak et al., 2015; FitzGerald et al., 2016; Sehmi et al., 2018). Expanding on this, benralizumab can also deplete basophils within peripheral blood in uncontrolled asthma (Eck et al., 2014), however a new study has shown that in mild asthmatics there is no depletion of peripheral or sputum basophils post-allergen provocation (Gauvreau et al., 2021). Although benralizumab uses an alternative approach of targeting the IL-5Rα, there still remains an incomplete ablation of exacerbation rates, similar to mepolizumab and reslizumab (Table 4).

#### 3.2.1 Possible Reasons for Suboptimal Responses With Anti-IL-5 Pathway Biologics

This brings to question why there is only a partial reduction in exacerbation rates in severe asthmatics treated with anti-IL-5RAα biologics. There are several reasons for sub-optimal or failure of response which will be described below. First, it is important to understand that severe asthmatics eligible for biologics are already on ICS therapy. We know from multiple reports that nonadherence to ICS in severe asthmatics is substantial (Gamble et al., 2009; Lee et al., 2018; Sulaiman et al., 2018), and that nonadherent patients receiving mepolizumab have worse clinical outcomes (d’Ancona et al., 2020). This brings up to two interesting propositions. The first is that worsened underlying disease as a result of noncompliance with ICS is associated with uncontrolled inflammation, and this may make it more difficult for biologics targeting eosinophils to have a noticeable clinical impact in patients with suboptimal responses. Secondly, it is important to emphasize that corticosteroids target other aspects of T2-high inflammation that are not inhibited by anti-IL-5 biologics, including IL-4 and IL-13 activity (Ray et al., 2016). One can postulate that if additional T2-high inflammatory pathways beyond the IL-5 pathway are kept under control in ICS-treated patients, then the beneficial effects of anti-IL-5 biologics may be amplified. Thus, continued and proper use of ICS is critical to dampening multiple T2-high endotypes that are not specifically targeted with anti-IL-5 biologics.

Now for a second proposal, there may be other inflammatory cells and cytokines, outside of the eosinophil-IL-5 pathway, that are equally, if not more important. For example, ILC2s, potent sources of IL-5, IL-9, IL-13, and PGD2, have been found to be higher in blood and sputum of severe asthmatics on high-dose steroids compared to mild asthmatics (Smith et al., 2016), and that these numbers are even higher in those with uncontrolled eosinophilia despite OCS. With respect to specifically targeting ILC2s, there have been a number of studies which have assessed the effect of corticosteroids and biologics on ILC2 numbers in severe asthma. Treatment with ICS has been shown to reduce ILC2-mediated inflammation, as well as ILC2 in nasal polyps, peripheral blood and sputum in asthma and asthma with allergic rhinitis (Walford et al., 2014; Yu et al., 2018). The corticosteroid-responsiveness of ILC2s may be dependent on activation by upstream cytokines including IL-33 and TSLP. This is supported by *in vitro* findings that IL-5 production from IL-33-induced ILC2s can be attenuated by corticosteroids, but not when ILC2s are stimulated by TSLP (Liu et al., 2018). Not unexpectedly, although anti-IL-5 agents reduce total sputum IL-5 levels, they do not attenuate ILC2s within sputum or blood, suggesting that these biologics neutralize IL-5 production from these cells but do not affect their overall function within the airways (Sehmi et al., 2018; Mukherjee et al., 2018b). Given the importance of ILC2s in asthma, there is a need to develop treatments that specifically target the function of these cells, and as *in vitro* studies have shown, this may be done most effectively at an upstream level with targeting of alarmins. Another consideration is that there are alternative signalling pathways, independent of alarmins, that can activate ILC2s, including the TNF superfamily pathway, including the TL1A/DR3 axis (Machida et al., 2020). Sputum TL1A levels are present in approximately 50% of prednisone-dependent severe asthmatics with uncontrolled eosinophilia (Machida et al., 2020). TL1A-induced activation of ILC2s, in the presence of TSLP and IL-2 is not responsive to dexamethasone (Machida et al., 2020). This further substantiates the heterogeneity of asthmatics, and highlights the hypothesis that the cytokine milieu within the airway determines responsiveness to corticosteroids and biologics. Aside from ILC2s, there are other important immune pathways to consider. For example, basophils have been shown to produce marked levels of IL-4 and IL-13 within the airways (Salter et al., 2015; Salter et al., 2016). Basophils are not only activated through an IgE-dependent pathway but also by alarmin cytokines including TSLP, IL-33, and IL-25 (Salter et al., 2015; Salter et al., 2016). Interestingly, in severe asthma, there is increased expression of receptors for IL-33 and IL-25 on basophils, particularly after IgE stimulation (Boita et al., 2018). Thus, alarmin cytokines can not only activate basophils in an IgE-independent manner, but IgE itself can upregulate receptor expression for alarmin cytokines, creating a vicious cycle. Although basophils have been found to express the IL-5 receptor, there is mixed evidence as to whether anti-IL-5 agents can affect basophil function. For example, Wright et al. (2019) found that 16 weeks of treatment with mepolizumab does not affect blood basophils in severe asthma. Similar to basophils, mast cells (MC) undergo extensive degranulation in fatal asthma suggesting that these cells are highly activated in severe asthma. Through both IgE-dependent and -independent mechanisms, MC not only release mediators such as histamine, prostaglandin and leukotrienes, but also produce a wide range of cytokines, including IL-4, IL-13, IL-6, IL-17, and TSLP (Bradding et al., 1992; Bradding et al., 1994; Ying et al., 1995; Ying et al., 2005). Collectively, there are numerous immune cells, outside of eosinophils, which can contribute to inflammatory processes inherent in severe asthma, independent of IL-5. Lastly, in addition to cytokines, there are granule proteins produced by eosinophils that play an important role in promoting airway obstruction. For example, EPX, a product of eosinophils, utilizes respiratory burst-derived H_2_O_2_ to generate reactive oxidants that can kill pathogens or activate airway cells (Wu et al., 2000). EPX has been shown to promote mucus plug formation by generating oxidants that modify mucins. Mucus plugging has been found in severe asthmatics and may predispose to infection (Dunican et al., 2018). Biologic therapy may not effectively target mucus plugging and is important to identify in patients with recurrent infections.

Other reasons for underlying suboptimal responses to biologics are related to dosing regimen and routes of administration. Mepolizumab has fixed dosing with SC administration versus reslizumab with IV weight-based dosing. Notably, the clinical benefits are the same for high and low doses of mepolizumab that is administered either IV or SC in severe asthmatics requiring high dose ICS (Mukherjee et al., 2018b). Conversely, severe asthmatics requiring daily prednisone have better clinical outcomes with higher doses of IV over lower doses of SC administration (Mukherjee et al., 2018b). These findings may be explained by lower doses administered SC not adequately neutralizing IL-5 within the airways, despite attenuated peripheral eosinophilia. For example, patients treated with 4 doses of weight-adjusted IV reslizumab after previously being treated with SC mepolizumab, resulted in suppressing both airway and peripheral eosinophilia (Mukherjee et al., 2018b). The magnitude of attenuation with reslizumab was greater compared to treatment with 12 doses of mepolizumab. The suppression of eosinophils and progenitors coincided with clinical improvement, as shown by increased FEV_1_ and asthma control. Patients who did not respond to anti-IL-5 treatment had higher sputum IL-5 levels. Collectively, this suggests that a greater concentration of anti-IL-5 either through increased dose or IV administration, is needed to neutralize T2-high inflammation, that is driven not only by eosinophils but also ILC2s, in peripheral and local airway compartments. Another hypothesis to consider is that low doses of anti-IL-5 biologics may cause worsening of airway eosinophilia through inducing immune-complex (IC) formation or complement consumption (Mukherjee and Nair, 2018; Mukherjee et al., 2020). ICs may act like cytokine depots, leading to increased potency of bound IL-5, resulting in worsening symptoms. We have seen detectable levels of sputum immunoglobulin-bound IL-5 in mepolizumab non-responders, coinciding with increased sputum IL-5. We only observed this immunoglobulin-IL-5 phenomenon in one patient receiving reslizumab, suggesting that this issue is prevented with higher dosing and/or IV delivery.

This leads us to the next hypothesis that inadequate dosing of IgG1 neutralising antibodies have the potential for disease worsening, particularly in those with an underlying airway autoimmune component. There is emerging evidence that local airway autoimmune responses contribute to corticosteroid insensitivity in severe asthma (Mukherjee et al., 2017; Mukherjee and Nair, 2018). A third of eosinophilic asthmatics have airway autoimmune responses, manifesting as auto-IgG antibodies directed against cell-derived granule proteins including, EPX (Liu et al., 2018). Severe asthmatics with sputum autoantibodies have characteristic clusters of sputum FEGs, indicative of active eosinophil cytolysis (Liu et al., 2018). Interestingly, we have shown that patients treated with reslizumab had a reduction in sputum anti-EPX IgG and antinuclear antibody (ANA), but not with mepolizumab. In fact, an increase in sputum anti-EPX IgG was seen in 66% of non-responders to mepolizumab. An additional study supports these findings, where 60% of severe asthmatics treated with mepolizumab showed a suboptimal response versus 32% with reslizumab (Mukherjee et al., 2020). In those who showed a poor response to mepolizumab, 23% worsened clinically, and had higher levels of sputum Anti-EPX IgG levels. An extension of this study showed that 43% of patients on either mepolizumab or reslizumab had suboptimal responses, with 14% of these patients having worsening asthma (Mukherjee et al., 2020). We found increased sputum IL-5, anti-EPX, EPX, and C3c in those with suboptimal response to biologics. In addition, increased C1-q/IgG levels and C1-q-IgG/IL-5-IgG dual-positive cells in sputum plugs were found in those who worsened on mepolizumab. This is supported by a case report where we described a severe asthmatic treated with mepolizumab had worsening of symptoms, and molecular analysis revealed increased anti-EPX and IL-5^+^ILC2s, suggesting that increased Th2 signaling leads to activation of IL-5-producing ILC2s and subsequent eosinophilia (Mukherjee et al., 2017). This brings up an interesting theory that inadequate drug dosing results in hetero immune-complex formation of complement-fixing antibody that is bound to the C1q molecule (Duncan and Winter, 1988), which can induce the complement cascade and promote recruitment of immune cells *via* the FcγR receptor (Stokol et al., 2004). As such, there may be an autoimmune-triggered IC-mediated phenomena in those with worsened response to mepolizumab. Finally, the monitoring of blood eosinophils did not help to identify this subgroup, nor did assessment of autoimmunity biomarkers. Peripheral eosinophilia was only observed in 8% of suboptimal responders, whereas 6% continued to have increased sputum eosinophils >3%, representing non-attenuated airway eosinophilia, and 69% of these patients had sputum eosinophils despite normal blood eosinophils. These findings support a discordance between blood and sputum eosinophils, and while peripheral eosinophils may be adequate for selecting patients that may benefit from anti-IL-5 therapy, it may be insufficient for monitoring therapeutic response to anti-IL-5 biologics (Mukherjee and Nair, 2015; Mukherjee et al., 2017; Mukherjee et al., 2018b; Drick et al., 2018; Ojanguren et al., 2018). Instead, we propose that sampling from the airways may be a more adequate way to identify monitor treatment response. For example, we have shown that sputum eosinophil count prior to treatment did not predict response to mepolizumab, nor was this the case with peripheral eosinophils. However, sputum eosinophils were effective for assessing response to treatment as early as 4 months post-treatment (Mukherjee et al., 2020). Of note, there is the emerging concept of “breathomics,” which is the phenotyping of patients through non-invasive identification of exhaled volatile organic compounds (VOCs) using gas chromatography and mass spectrometry (De Vries et al., 2018; Sterk, 2019). Measurement of VOCs has been shown to have similar accuracy to sputum cell counts and FeNO (Sterk, 2019). This may be an alternative or additive approach to monitoring treatment response to biologic agents.

There is a subset of severe asthmatics with frequent respiratory infections that is thought to be secondary to underlying airway neutrophilia. There are higher levels of IgM and IgG in asthmatics compared to healthy controls with recurrent respiratory tract infections (Ho et al., 2020). Specifically, eosinophilic asthmatics have lower levels of IgA compared to healthy controls. We have shown that administration of IVIg leads to increased total IgG and subtypes, and these patients had fewer infective exacerbations over 12 months (Ho et al., 2020). These findings suggest that although eosinophilic inflammation may be dampened by biologics, exacerbations continue to occur due to underlying neutrophilia and humoral deficiency. It is important to consider assessing immunoglobulin levels in those with frequent asthmatics and replace if necessary. We have previously assessed severe asthmatic responses over 14 months to benralizumab, and found that 27% of patients had suboptimal responses and 40% of these patients had worsening disease (Poznanski et al., 2021). Only two patients with worsening asthma had sputum eosinophilia, whereas 16 had evidence of infective exacerbation with neutrophilic inflammation. A suboptimal response to benralizumab has been proposed to be due to impaired NK function and/or number (Poznanski et al., 2021). Overall, respiratory infections increased with benralizumab and had associated sputum neutrophilia, which is in contrast to mepolizumab or reslizumab that is associated with eosinophilic exacerbations. Previous history of infections predicted poor responses to benralizumab (Poznanski et al., 2021). Lastly, benralizumab is seemingly more potent than the other anti-IL-5 agents at suppressing airway eosinophilia. Although there have been no head-to-head trials with anti-IL-5 agents, benralizumab appears to be non-superior relative to mepolizumab or reslizumab from a clinical standpoint. As mentioned earlier, there are numerous other pathways, aside from the IL-5-eosinophil pathway which may contribute to asthma pathogenesis but may not be adequately attenuated by IL-5-targeted biologics. For example, alternative Th2 cytokines, such as IL-4 and IL-13 or alarmin cytokines, and other immune cells such as basophils, Th2 cells, MC, and ILC2s may still be present and activated within the airways despite treatment with benralizumab. Thus, regardless of complete attenuation of eosinophils, there are other, redundant pathways that can carry out airway inflammation. The combination of anti-IL-5 agents with biologics that target other important Th2 pathways may confer better clinical outcomes, however this needs to be studied in more detail in the future.

### 3.3 IL-4/IL-13 Targeted Therapy

In order to understand the efficacy of IL-4/IL-13 agents, it is important to understand receptor signaling involved with these two cytokines. IL-13 signals through the IL-13 receptor, of which there are two subtypes, including IL-13Rα1 and IL-13Rα2. IL-13Rα1 binds to IL-13 with low affinity, but when the IL-4 receptor, IL-4Rα1, joins to form a heterodimer, IL-13 is bound with greater affinity. The IL-13Rα2, binds to IL-13 with high affinity but lacks a cytoplasmic domain thus does not signal downstream, however it may act as a negative regulator of IL-13 and IL-4 signaling. While isolated blockade of either IL-4 or IL-13 has not been shown to be effective in treatment of severe asthma, dual blockade of IL-4 and IL-13 has shown promise.

With respect to anti-IL-13 biologics, two agents have been studied, lebrikizumab and tralokinumab. Studies looking at moderate-severe asthma with T2-high inflammation (total IgE ≥100 IU/ml and blood eosinophils ≥140 cells/μl) have shown that treatment with lebrikizumab resulted in 60% reduction of AAER and improved FEV_1_, but no effect on symptoms (Corren et al., 2011). Subgroup analysis showed that patients with higher serum periostin (≥50 ng/ml) or FeNO had greater improvement in lung function. Furthermore, there was an observed decrease in FeNO and serum IgE, but not eosinophils. Larger RCTs have shown that severe asthmatics with T2-high biomarkers (serum periostin ≥50 ng/ml and/or blood eosinophils ≥300 cells/μl) treated with lebrikizumab had reduced AAER but no coinciding improvement in symptom scores and only marginal improvement in FEV_1_ (Hanania et al., 2015; Hanania et al., 2016). Similarly, the majority of studies with tralokinumab did not show promising clinical outcomes (Piper et al., 2013; Brightling et al., 2015; Panettieri et al., 2018; Russell et al., 2018). Collectively, anti-IL-13-specific agents are not effective in treating severe asthma. This may be due to IL-13 primarily being involved with AHR as opposed to exacerbation and/or inflammation. With respect to tralokinumab, it targets both the IL-13Rα1 and IL-13Rα2 subunits, and thus may dampen the anti-inflammatory effect through IL-13Rα2 (Table 5).

The trials with respect to anti-IL-4 biologics have also been disappointing. Pascolizumab was shown to be well tolerated in animal studies with monkeys and effective in neutralizing bioactivity of IL-4 (Hart et al., 2002). Human RCTs have shown that treatment with Altrakincept, a nebulized anti-IL-4 agent, in moderate-severe asthmatics significantly improved in FEV_1_ and reduced FeNO, with no effect on AAER (Borish et al., 1999; Borish et al., 2001). Overall, anti-IL-4 agents have not yielded sufficient clinical efficacy to warrant further investigation and research has been halted. The lack of efficacy, may in part, be due to redundancy provided by IL-13, which signals through the same heterodimer.

Given the lack of impressive data from anti-IL-4 and IL-13 individual biologic agents, it was thought that perhaps targeting a common pathway between both cytokines may yield greater effect. Dupilumab is the first dual IL-4/IL-13 biologic approved for asthma treatment. It targets the shared IL-4Rα receptor and thus blocks signalling of both IL-4 and IL-13. An initial trial with dupilumab treatment in eosinophilic asthmatics (blood eosinophils ≥300 cells/μl or sputum eosinophils ≥3%) resulted in an 87% reduction in AAER (Wenzel et al., 2013). Of note, the treatment groups were instructed to stop their maintenance LABA at week 4 and wean from ICS from weeks 6–9. Exacerbations were only seen after the point of inhaler withdrawal, suggesting that dupilumab may be acting on the same pathway as ICS/LABA inhalers and hence has a redundant effect. A larger RCT with severe asthmatics (blood eosinophils ≥300 cells/μl) over a 24-week period showed 81% reduction in AAER compared to 60% reduction with the low eosinophilia group, both of which were significant compared to placebo. These findings suggest that dupilumab may be effective regardless of eosinophilic status. However, other RCTs showed that dupilumab treatment only yielded significant reduction in AAER in patients with blood eosinophils ≥150 cells/μl and FeNO ≥25 ppb. In terms of biomarkers to monitor response, dupilumab induces transient increases in blood eosinophils but significant reduction in FeNO, suggesting that FeNO may be a better biomarker to assess treatment eligibility and efficacy for this treatment.

#### 3.3.1 Possible Reasons for Suboptimal Responses with Anti-IL-4/IL-13

There have been some case reports of adverse events when switching from anti-IL-5 to anti-IL-4/IL-13. For example, patients switched from anti-IL-5 to anti-IL-4/IL-13 had worsening in asthma control and showed increased use of OCS, with substantial increases in peripheral eosinophils (Eger et al., 2021). The reason for this worsening is not entirely understood, but the working hypothesis is that these patients had underlying anti-neutrophil cytoplasmic antibody (ANCA) negative EGPA triggered by the rebound hypereosinophilia brought on by dupilumab. We propose that both pathways (IL-5, IL-4/IL-13) will need to be targeted to allow for optimal disease control. Unfortunately, no studies thus far have looked at the efficacy of combining anti-IL-4/IL-13 and anti-IL-5 biologics for asthma treatment.

Sub-optimal responses to anti-IL-4/IL-13 may be explained by these agents primarily focusing on reducing AHR as opposed to dampening airway inflammation (Gour and Wills-Karp, 2015). We propose that dupilumab should be used in patients who have symptoms of AHR, and if there is overlap with airway inflammation, it may be reasonable to pair with an upstream inhibitor, such as an anti-IL-33 or anti-TSLP agent. In addition, it is well known that mucus hyperplasia is promoted by the IL-4/IL-13 axis (Munitz et al., 2008; Bao and Reinhardt, 2015) and as such, patients with mucus hypersecretion as a primary symptom should be managed with agents targeting this axis such as dupilumab.

Interestingly, Wechsler et al. assessed the treatment of severe asthmatics with a combination of dupilumab and anti-IL-33 (Wechsler et al., 2020a). The anti-IL-33 biologic on its own was able to improve asthma control and lung function, but this was not synergistic when combined with dupilumab. This may have been due to both agents having redundant T2-high pathways, whereas there was insufficient targeting of IL-5 or TSLP, and hence continued activity of ILC2s and Th2 cells. Alternatively, this study was not adequately powered for between group comparisons. This study certainly provides food for thought and warrants further investigation with respect to studying combined biologic regimens.

### 3.4 Alarmin Cytokine Therapy

The development of anti-alarmin biologics has been one of the most exciting innovations in asthma therapy to date. Tezepelumab is a human IgG_2_ antibody directed against TSLP, that can be administered IV or SC. The first landmark trial to assess the efficacy of tezepelumab was carried out in mild asthmatics, which showed significant improvement in FEV_1_ and reduction of peripheral and sputum eosinophils, along with decreased FeNO levels during the late phase response post-allergen provocation (Gauvreau et al., 2014). Subsequent RCTs in severe asthmatics showed that tezepelumab treatment resulted in a 44–71% reduction in AAER, irrespective of baseline peripheral eosinophilia (Table 6) (Marone et al., 2019; Corren et al., 2021a; Menzies-Gow et al., 2021; Sverrild et al., 2021). Patients were also shown to have improvements in FEV_1_ and symptoms, with a coinciding reduction in peripheral eosinophils and serum IgE. Collectively, these data suggest that targeting upstream cytokines, such as TSLP, may prove to be beneficial in multiple asthma endotypes, both within and outside of T2-high inflammation.

Initial studies that looked at anti-IL-33 agents were done in other sites of allergy beside asthma, specifically in atopic dermatitis and peanut allergy. Etokimab has been shown to improve symptoms related to atopic dermatitis and reduce desensitization to peanuts (Chen et al., 2019; Chinthrajah et al., 2019). These clinical findings were associated with significant reductions in peripheral eosinophils and T2 cytokine levels, along with total serum IgE. There are multiple RCTs underway assessing the efficacy of anti-IL-33 biologics to treat asthma, but most results are pending (Table 7). Of note, the first RCT was recently published in 2021 by Wechsler et al. which assessed the effect of an anti-IL-33 agent called itepekimab in a phase 2 trial with moderate-to-severe asthma (Wechsler et al., 2021). They found that following 12 weeks of treatment with itepekimab there was an improvement in asthma control and quality of life. Furthermore, loss of asthma control occurred in 22% of patients in the itepekimab group compared to 19% in the dupilumab group and 27% in the combined group. Interestingly, itepekimab alone, or in combination with dupilumab resulted in decreased blood eosinophil. Itepekimab alone was also able to reduce FeNO, serum total IgE, periostin, and plasma eotaxin-4 but to a lesser extent compared to dupilumab or combined therapy. Overall, this anti-IL-33 agent reduced blood eosinophils to a lesser extent than anti-IL-5 agents and it had reduced effect on eotaxin-3, which is an IL-13 product, compared to dupilumab. These findings suggest incomplete inhibition of Th2 inflammation with blockade of IL-33, given that other alarmin cytokines are still active, including TSLP. More studies are needed to determine the effect of anti-IL-33 agents on airway eosinophils and other inflammatory parameters.

Overall, there have been not been sufficient RCTs with anti-alarmin agents to be able to identify suboptimal or failure to respond and what might be responsible for these outcomes. It will be helpful in the future to assess dual targeting of upstream and downstream inflammatory cytokines to treat severe asthma.

### 3.5 Other Current Therapy

There have been other agents that target various aspects of eosinophil function which have been assessed, albeit less extensively than the aforementioned biologics (Table 8). Sialic-acid-binding immunoglobulin-like lectin (Siglec)−8 is a cell surface receptor found on mast cells and eosinophils. Initial studies have shown that an anti-Siglec-8 induces death of cytokine-primed eosinophils and inhibits IgE-mediated mast cell activation (Levine et al., 2020). RCTs with AK002 in severe allergic conjunctivitis reported improved symptoms (Levine et al., 2020), and these patients were also found to have comorbid asthma, with 72% reduction in asthma symptom scores. Hirano et al. assessed AK002 in eosinophilic gastritis and esophagitis, showing significant improvement in dysphagia symptoms and reduction in esophagus eosinophils (Table 8) (Hirano et al., 2020). Further studies are needed to determine the efficacy of anti-Siglec-8 in asthma.

**TABLE 8:** Summary of other therapeutic targets in severe asthma.

Other agents						
Landmark study and year	RCT phase	Disease severity	Disease phenotype	Dosing, duration and route of administration	Clinical effect	Molecular effect
KRONOS, 2020 Levine et al. (2020)	Phase 1b	Severe allergic conjunctivitis	Atopic	Biologic: AK002	- ACQ score improved by 74% vs. placebo	-N/A
Anti-Siglec 8				Dose: 0.3, 1, 3 mg/kg q4W	- 72% reduction in asthma sx	
				Route: IV		
				Duration: 6 M		
Hirano et al. (2020)	Phase 2	Eosinophilic gastritis and esophagitis	Atopic	Biologic: AK002	- Improvement in dysphagia symptom scores	- Reduction in esophageal eosinophils
Anti-Siglec 8				Dose: 0.3, 1, 3 mg/kg q4W		
				Route: IV		
				Duration: 4 M		
EXHALE, 2017 Prussin et al. (2017)	Phase 2	Moderate-severe asthma	≥300 cells μl PB eosinophils	Dose: 75–300 mg/day	- Improved Pre-BD FEV_1_ from baseline	- Reduced PB eosinophils at 12 W
Dexpramipexole				Route: PO		
				Duration: 12 W		
LUSTER 1&2, 2021 Brightling et al. (2021b)	Phase 3	Severe Asthma	≥250 or <250 cells μl PB eosinophils	Dose: 150–450 mg/day	- Improved AAER in eosinophil high patients	- N/A
Fevipiprant				Route: PO		
				Duration: 52 W		
Gonem et al. (2016)	Phase 2	Moderate-Severe Asthma	≥2% SP eosinophils	Dose: 250 mg BID	- Favourable safety profile	- 4.5 times reduction in SP eosinophils
Fevipiprant				Route: PO		
				Duration: 12 W		
Bateman et al. (2017)	Phase 2	Moderate-Severe Asthma	IgE ≥0.35 IU mEq	Dose: 1–450 mg OD or BID	- Improved pre-BD FEV_1_ at 12 W	- N/A
Fevipiprant				Route: PO		
				Duration: 12 W		
Brightling et al. (2021a)	Phase 2	Severe asthma		Dose: 90 mg q4W	- Shorter time to asthma worsening	- No change in blood eosinophils, sputum eosinophils or neutrophils
Risankizumab				Route: SC		
				Duration: 24 W		

Dexpramipexole is a small molecule traditionally developed as treatment for amyotrophic lateral sclerosis. These studies showed significant and persistent reduction in blood eosinophils 1–2 months after drug initiation. The spotlight has now turned to assessing the use of dexpramipexole for asthma and CRS. Initial studies with CRS have shown that dexpramipexole can reduce peripheral eosinophils to <0.020 × 10^9^/L at month 6 post-initiation, and yield a 97% reduction in nasal polyp tissue eosinophils from baseline (Laidlaw et al., 2019). Similarly, another study showed that dexpramipexole can reduce blood eosinophils by 93% compared to baseline at month 6 and 94% in tissue nasal polyps (Prussin et al., 2017). In terms of asthma, 12 weeks treatment with dexpramipexole resulted in significant reduction of peripheral eosinophils and improved FEV_1_ (Prussin et al., 2021). More data is needed to determine effect on sputum eosinophils and whether this reduction translates into meaningful clinical outcomes.

Prostaglandin D2 receptor 2 (PGD2) is a potent mediator involved in asthma pathogenesis with the main function to promote airway smooth muscle contraction. In addition, PDG2 activates the DP2 chemokine receptor, also known as CRTH2, on Th2 cells, ILC2s, granulocytes, and monocytes. The PGD2/CRTH2 axis is implicated in cell adhesion, survival, and activation resulting in cytokine/chemokine production, and subsequent downstream eosinophilia. Fevipiprant is an oral PGD2 receptor antagonist recently developed for asthma. Studies have shown that in moderate-severe asthma fevipiprant reduces sputum and bronchial submucosal eosinophils, and reduces airway smooth muscle mass compared with placebo (Saunders et al., 2019). Similar studies have shown that fevipiprant can induce a 3–5 greater 3-5 fold reduction in sputum eosinophils (Gonem et al., 2016; Saunders et al., 2019). From a clinical perspective, treatment with fevipiprant can improve pre-dose trough FEV_1_ and symptom scores, as well as reduce AAER (Erpenbeck et al., 2016). Conversely, other trials did not show improvement in symptom scores or FEV_1_ (Castro et al., 2021). Overall, findings have been inconsistent with respect to clinical efficacy of fevipiprant treatment. A recent systematic review confirms this by showing that although the agent is safe, it does not reach minimal clinically important difference (Yang et al., 2021). Suboptimal responses may be explained by the PGD2/CRTH2 axis being one of the many pathways that control Th2 and ILC2 activation, and that anti-alarmin pathways may be more crucial to target. In summary, there is a need for more studies determining the efficacy of combining these agents with other biologics.

Lastly, IL-23 has been implicated in airway inflammation that is mediated by Th2 and Th17 cytokines. Animal models have shown IL-23 to promote Th17 cell proliferation, which in turn maintains IL-17A and IL-17F production and neutrophil recruitment (Li and Hua, 2014). IL-23 also promotes Th2 cytokine production and eosinophil infiltration (Wakashin et al., 2008). Brightling et al. (2021a) recently conducted a phase 2a RCT assessing the effect of an anti-IL-23p19 agent, called risankizumab in severe asthma. They found that the time-to-first asthma worsening and rate ratio for annualized asthma worsening was shorter in the risankizumab group compared to placebo. There was no effect of risankizumab on FeNO, median blood eosinophil count or sputum eosinophils and neutrophils. They did, however, report that risankizumab reduced the sputum IL-23 gene set and pathways associated with activation of cytotoxic T cells and NK cells. Their findings challenge the proposed role of Th17 and IL-23 in severe asthma. The worsening in asthma control may have been attributed to targeting of IL-23 leading to an increase in Th2 mediators, such as IL-13, thereby resulting in increased smooth-muscle tone within the airways. Interestingly, the poorer outcomes in the risankizumab group were amplified in those with higher blood eosinophils counts. Given the evidence of potential harm related to IL-23 blockade, it may be reasonable to steer away from this area of study or consider studies assessing the efficacy of combining anti-IL-23 or anti-IL-17 agents with Th2-targeted agents.

## 4 Conclusion

Overall, we have described a number of mechanisms that could be contributing to failed or sub-optimal response to biologic therapy in severe asthma. The main themes are the need for proper dosing and route of administration of biologics, the identification of underlying inflammation through proper immune endotyping, as well as the targeting of multiple pathways, both upstream and downstream. An astute understanding of the molecular mechanisms and their associated clinical manifestations require careful consideration for development of valid biomarkers that will help guide optimal treatment and monitor therapeutic response. Moving forward, we need these biomarkers to assess individual patient symptoms and determine the underlying immunological mechanism that may be primarily responsible for driving disease severity and aid in choosing the right targeted therapy.
